# Supplementary material for: Influences on anticipated time to ovarian cancer symptom presentation in women at increased risk compared to population risk of ovarian cancer
Source: BMC Cancer. 2017 Dec 4;17:814. doi: 10.1186/s12885-017-3835-y (PMC5716383; doi:10.1186/s12885-017-3835-y)
Supplement: Supplementary file 1 — Correlation matrix for variables in the structural equation models. Description of data: correlation matirx, means and standard deviations for variables in the three structural equation models. (DOCX 20 kb) [file 12885_2017_3835_MOESM1_ESM.docx]

**Influences on anticipated time to ovarian cancer symptom presentation in women at increased risk compared to population risk of ovarian cancer**

Stephanie Smits^1§^, Jacky Boivin^2^, Usha Menon^3^, Kate Brain^1^

^1^Division of Population Medicine, School of Medicine, Cardiff University, Cardiff, UK

^2^School of Psychology, Cardiff University, Cardiff, UK

^3^Women's Cancer, Institute for Women's Health, University College London, London, UK

^§^Corresponding author: Division of Population Medicine, School of Medicine, Cardiff University, Neuadd Meirionnydd, Heath Park, Cardiff, CF14 4YS. Tel +44(0)2920 687156, Fax: +44(0)2920 687 219, Email: smitsse@cardiff.ac.uk

Authors two and four contributed equally to this paper.

Author email addresses: Jacky Boivin ([boivin@cardiff.ac.uk](mailto:boivin@cardiff.ac.uk)), Usha Menon ([u.menon@ucl.ac.uk](mailto:u.menon@ucl.ac.uk)), Kate Brain (brainke@cardiff.ac.uk)

**Abstract**

**Background**: In the absence of routine ovarian cancer screening, promoting help-seeking in response to ovarian symptoms is a potential route to early diagnosis. The factors influencing women’s anticipated time to presentation with potential ovarian cancer symptoms were examined.

**Methods:** Cross-sectional questionnaires were completed by a sample of women at increased familial risk (n=283) and population risk (n=1043) for ovarian cancer. Measures included demographic characteristics, symptom knowledge, anticipated time to symptom presentation, and health beliefs (perceived susceptibility, worry, perceived threat, confidence in symptom detection, benefits and barriers to presentation). Structural equation modelling was used to identify determinants of anticipated time to symptomatic presentation in both groups.

**Results**: Associations between health beliefs and anticipated symptom presentation differed according to risk group. In increased risk women, high perceived susceptibility (r=.35***), ovarian cancer worry (r=.98**), perceived threat (r=-.18**), confidence (r=.16**) and perceiving more benefits than barriers to presentation (r=-.34**), were statistically significant in determining earlier anticipated presentation. The pattern was the same for population risk women, except ovarian cancer worry (r=.36) and perceived threat (r=-.03) were not statistically significant determinants.

**Conclusions**: Associations between underlying health beliefs and anticipated presentation differed according to risk group. Women at population risk had higher symptom knowledge and anticipated presenting in shorter time frames than the increased risk sample. The cancer worry component of perceived threat was a unique predictor in the increased risk group. In increased risk women, the worry component of perceived threat may be more influential than susceptibility aspects in influencing early presentation behaviour, highlighting the need for ovarian symptom awareness interventions with tailored content to minimise cancer-related worry in this population.

*Keywords:* ovarian cancer, symptom awareness, symptom presentation, health beliefs, increased risk

# Background

Once described as ‘the silent killer’ [[1](#_ENREF_1), [2](#_ENREF_2)] ovarian cancer is now recognised as having identifiable symptoms that are present at all stages of the disease [[2](#_ENREF_2)]. The importance of symptom awareness in the early diagnosis of cancer has been highlighted through the UK National Awareness and Early Diagnosis Initiative and the American Cancer Society guidelines for early detection [[3](#_ENREF_3), [4](#_ENREF_4)]. Ovarian cancer symptoms are vague and poorly differentiated from other common conditions [[5](#_ENREF_5)], and are often misattributed to ageing, the menopause, or stress [[6-8](#_ENREF_6)]. Understanding the determinants of anticipated ovarian symptomatic presentation (how long it would take to present to a doctor if they thought they were experiencing a symptom) is important because ovarian cancer screening is not yet proven or routinely available for women in the general population or those at increased risk due to a family history of [breast/ovarian] cancer or gene mutations [[9](#_ENREF_9), [10](#_ENREF_10)]. Understanding the determinants of anticipated symptomatic presentation is beneficial, with much to be gained from the early detection of ovarian cancer, such as improved treatment options and better survival outcomes [[11](#_ENREF_11)]. While a number of studies have examined cancer knowledge and symptom presentation in the general population [[12-14](#_ENREF_12)], few studies have been conducted involving women at increased risk for ovarian cancer. In the general population, low levels of ovarian cancer symptom knowledge have been reported [[15](#_ENREF_15)], as well as a reported lack of association between awareness of gynaecologic cancer symptoms and anticipated presentation behaviour [[16](#_ENREF_16)]. It could be expected that the increased saliency of cancer risk would lead to earlier symptom presentation in women at increased risk; however, empirical evidence is lacking regarding the influences on symptom presentation in women at increased risk compared to the general population.

The Health Belief Model (HBM) [[17](#_ENREF_17)] can be used to explore the determinants of anticipated symptomatic presentation. The HBM proposes that two variables directly influence likelihood of anticipated presentation behaviour: (1) perceived threat, and (2) the belief that the benefits of carrying out the action outweigh the barriers. According to the HBM, worry may be related to perceived health threat [[18-20](#_ENREF_18)], which is the combination of perceived susceptibility and perceived severity [[17](#_ENREF_17), [21](#_ENREF_21)]. A review of empirical literature on the role of cancer worry in screening uptake, and the theoretical approaches to understanding of worry suggested that worry is an emotional representation of susceptibility or severity [[22](#_ENREF_22)]. Evidence suggests higher perceived threat increases the likelihood of engaging in behaviour that is likely to manage or reduce this threat [[17](#_ENREF_17)]. Cancer-related worry appears to be a strong influence on health-related decision making for women at increased risk [[23](#_ENREF_23), [24](#_ENREF_24)], with high levels of ovarian cancer worry and perceived susceptibility [[25](#_ENREF_25), [26](#_ENREF_26)] predicting higher ovarian screening uptake [[23](#_ENREF_23)]. Given the impact of risk and associated worry on cancer screening uptake, the impact of risk and worry on symptom presentation behaviour needs to be considered [[27](#_ENREF_27)]. If high levels of cancer worry are contributing to presentation in these women, it is important to identify the mechanisms through which worry can be reduced or managed. Increased risk women may demonstrate a different pattern of health beliefs in relation to ovarian cancer symptomatic presentation compared to women at population risk. Research is therefore needed to explore and compare these two populations, and to increase understanding of the determinants of anticipated presentation, particularly the potential role of emotions such as cancer-related worry [[28](#_ENREF_28)]. In ovarian cancer, there is currently no agreed definition of an optimal symptom presentation interval. However, as early stage at diagnosis is associated with better treatment outcomes and ultimately, survival [[11](#_ENREF_11), [29](#_ENREF_29)], early presentation is considered advantageous [[4](#_ENREF_4)].

Structural equation modelling (SEM) will be used to test the HBM model and to identify correlates of anticipated presentation. SEM is a statistical technique that allows for the simultaneous test of multiple causal relations [39] and can be used to test theoretical models, such as the HBM in novel health contexts. SEM is an advantageous method as it allows for a deeper exploration of the relationships between variables than standard regression analysis. Particularly, SEM allows for theoretical models to be tested, for simultaneous analysis to be conducted, and for latent (unobserved) variables to be modelled.

The present study was undertaken to examine determinants of anticipated time to presentation for potential ovarian cancer symptoms in women at increased risk, with a population risk comparison group. Women at increased risk were hypothesised to differ from women at population risk in terms of health beliefs, including higher levels of worry, knowledge, perceived susceptibility, perceived threat, benefits and barriers to presentation, a greater degree of personal experience of ovarian cancer, and earlier anticipated time to symptom presentation.

# Methods

***Participants and Procedures***

Recruitment and study procedures were different for the increased risk and population risk women and are presented separately. The study received ethical approval from Cardiff University, School of Medicine.

*Increased risk sample*

Participants were recruited from a database of 1,999 women who had previously been identified as increased risk of ovarian cancer based on their family history or genetic test results, and who had taken part in a psychological evaluation of familial ovarian cancer screening (PsyFOCS) study [[30](#_ENREF_30)]. High risk women have at least a 10-15% lifetime risk of ovarian cancer, compared to 1.3% in women at population risk [[31](#_ENREF_31)]. Of the PsyFOCS sample, 446 registered interest in taking part in a further study on ovarian symptom awareness. In addition, a further 29 participants were recruited via the UK based charity Ovacome (http://www.ovacome.org.uk). Women who had registered interest in the study were invited to complete a postal or online questionnaire, according to their preference. Selection criteria for the present study included the ability to give informed consent, not having a previous diagnosis of ovarian cancer, or a procedure to remove one or both ovaries. In total 164 (34.5%) did not return the questionnaire and 28 (5.9%) were excluded due to previous oophorectomy. The final sample was n=283 (63.3%). Of these, 29 were from Ovacome (10.2%) and the remaining 254 (89.8%) from the PsyFOCS recruitment pool. Four participants completed the electronic version of the survey. The mean age of the women was 52.87 years (SD=10.40), with most having completed secondary education or above (71.0%, n=201) (see Table 1).

*Population risk sample*

Women from the general population were recruited in Wales as part of the International Cancer Benchmarking Partnership [[28](#_ENREF_28)]. Random probability sampling was used to achieve a population-representative sample using electronic telephone directories as the sampling frame. Where more than one person was eligible, the Rizzo method was used to randomly select one person to be interviewed, thereby giving an equal chance of selection to all eligible people living in the household [[32](#_ENREF_32)]. Computer assisted telephone interviews were completed by 1,043 women. The selection criteria included women aged over 50 years, residing in Wales with the ability to give informed consent, not having had a previous diagnosis of ovarian cancer and not having had a procedure to remove one or both ovaries [[28](#_ENREF_28)]. Due to the sampling method used for the general population (random digit dialling), it is not possible to estimate the number of eligible participants [[28](#_ENREF_28)] (Brain, Smits et al. 2014). Of the 1385 female respondents, 315 were excluded due to a personal history of ovarian cancer or having had a procedure to remove one or both ovaries. The final sample comprised 1043 women. As shown in Table 1, the mean age of the women was 64.53 (SD=9.49) and the majority had completed education up to age 16 (55.8%, n=570). The increased risk sample was significantly younger, more likely to be married or cohabiting, and to have a higher educational level.

**Health Belief Model Measures**

***Individual perceptions***

Two measures were used to assess individual perceptions. *Perceived susceptibility* was measured by asking “Compared to most other women your age, how likely do you think it is that you will get ovarian cancer at some time in your life?” Responses were rated from 1 (much less likely) to 5 (much more likely) (adapted from [[33](#_ENREF_33)]. *Ovarian cancer worry* was measured with the Ovarian Cancer Worry Scale [[34](#_ENREF_34)], which is an adaptation of the Cancer Worry Scale [[35](#_ENREF_35)]. The Ovarian Cancer Worry Scale consists of three questions, which assess frequency of worry, the impact this has on mood and the impact on daily functioning, each on a 5 point scale giving a range of 3-15 (Cronbach’s α = 0.80 for the increased risk sample, α = 0.69 for the population risk sample).

***Modifying factors***

Eleven statements assessed ovarian cancer symptom *knowledge*, and were adapted from the validated ovarian cancer awareness measure [[36](#_ENREF_36)] and included less common symptoms to reflect the UK Department of Health’s ‘Key Messages’ on ovarian cancer for health professionals and the public [[37](#_ENREF_37)]. The 11 symptoms were: a persistent pain in the abdomen, a persistent pain in the pelvis, vaginal bleeding after the menopause, persistent abdominal bloating, increased abdominal size on most days, not wanting to eat because feel persistently full, difficulty eating usual amounts of food on most days, passing more urine than usual, a change in bowel habits, extreme tiredness and back pain. Scores were summed to give a total knowledge score (range 0-11). *Confidence in symptom detection* was assessed by confidence in symptom detection by asking “How confident are you that you would notice a symptom of ovarian cancer?” Scores ranged from 1 (not at all) to 4 (very confident) [[36](#_ENREF_36)].

***Cues to action***

*Personal experience* with ovarian cancer was assessed through the question “Have you, or any friends or family members that are close to you, ever been diagnosed with ovarian cancer?” Participants who responded ‘yes - self’ were excluded. Response options were coded as 0= no ovarian cancer experience, 1= ovarian cancer experience.

***Likelihood of action***

Eleven items were used to develop scales for perceived benefits and percieved barriers [[13](#_ENREF_13), [36](#_ENREF_36)]. For eight items participants were asked to “indicate whether any of the following might put you off going to the doctor if you thought you had a symptom of ovarian cancer” (e.g. “I would be too scared”). The response options were: yes often (code =3), yes sometimes (code = 2) and no (code =1). For the remaining three items, participants were asked “please indicate how much you agree or disagree with each statement” (e.g. “If found early, ovarian cancer can often be cured”) rated from 1 (strongly disagree) to 4 (strongly agree).

***Likelihood of behaviour***

*Anticipated presentation* was measured by asking “If you had a symptom that you thought might be a sign of ovarian cancer, how long would it take you to go to the doctors from the time you first noticed the symptom?”[[36](#_ENREF_36)]. Response options were: I would go as soon as I noticed, up to one week, over one week up to two weeks, over two weeks up to three weeks, over three weeks up to four weeks, and more than a month. Responses were re-coded as ‘0 = I would go as soon as I noticed, no delay’ and ‘1 = any delay, between up to a week to more than a month’.

***Data analysis***

Data were examined to determine suitability for analyses. Screening identified one participant reporting a score of 15 for ovarian cancer worry (sample mean = 6.15, sd= 1.94) and this outlier case was removed from analysis. The total increased risk sample (n=283) and population risk sample (n=1,043) were combined (n=1,326) for an overall test of the structural relations in the SEM test of the Health Belief Model (HBM). However, sample profile characteristics and descriptive statistics were presented separately for the two groups. In preliminary work to generate measures for the study, separate principal components analyses of HBM items were conducted for the two groups in order to identify the salient factors contributing to the HBM scales for each risk group.

*Increased risk sample:* Four factors were extracted which explained a total 63.12% of the variance. The factors were labelled Perceived Barriers (26.60% of variance, eigenvalue 3.19, Cronbach’s α=0.72, range 6-8, mean score 7.94, sd=2.17), Perceived Benefits (16.42% of variance, eigenvalue 1.97 Cronbach’s α= 0.81, range 3-12, mean score 10.04, sd=1.90), Fear (11.57% of variance, eigenvalue 1.39 r=0.72, p<0.001) and Perceived Susceptibility (8.52%, eigenvalue 1.02). Fear referred to fear of what might be discovered and therefore for the purpose of SEM, the factor-derived scales for fear and perceived barriers were combined to create a perceived barriers construct (8 item scale Cronbach’s α= .75) in order to create the perceived barriers item for the likelihood of action component of the HBM. As the HBM defines the likelihood of action as perceived benefits minus perceived barriers, these calculations were made in SPSS (version 18) creating the likelihood of action scale that ranged from -15 to 4. Scores at the negative end of the Likelihood of Action scale represented more perceived barriers than benefits, and scores at the positive end of the scale indicated more perceived benefits than perceived barriers.

*Population risk sample:* Four factors were extracted which explained 57.84% of the variance. The factors were labelled Emotional Barriers (23.96% of variance, eigenvalue 2.88, Cronbach’s α =0.67, range 5-15, mean score 6.02, sd=1.65), Practical Barriers (15.71% of variance, eigenvalue 1.89, Cronbach’s α=0.59, range 3-9 mean score 3.51, sd=1.02), Perceived Benefits (9.78, eigenvalue 1.17, Cronbach’s α= 0.68, range 3-12 mean score 10.94, sd=1.29) and Perceived Susceptibility (8.39% of variance, eigenvalue 1.01). Similarly to the increased risk group, the scales for emotional barriers and practical barriers were combined to create a perceived barriers construct for use in SEM (8 item scale Cronbach’s α= .72). Factor analysis of the 12 HBM constructs showed the same pattern of underlying constructs for the two risk groups, with the exception of the perceived barriers constructs that were created based on the principal components analysis for each group for the purpose of SEM, where minor item differences were observed. For the increased risk group, the extracted factors for perceived barriers differentiated fear of the discovery of ovarian cancer, with the perceived barriers constructed that was created for the purpose of SEM consisted of a combination of fear and perceived barriers items as identified in the principal components analysis. For the population risk group the extracted factors differentiated practical barriers involving time constraints, with the perceived barriers constructed that was created for the purpose of SEM consisting of a combination of emotional and practical barriers.

Prior to SEM analysis, a measurement model was created in order to observe whether perceived susceptibility and ovarian cancer worry were part of the same trait complex of perceived threat. The measurement model consisting of perceived susceptibility, ovarian cancer worry and the latent variable perceived threat can be seen embedded in the structural model in Figure 2. The other HBM components were then added in order to create the full structural model. The full SEM examined relations between this latent threat complex and other constructs. Specifically, a SEM was computed investigating whether individual perceptions, modifying factors, perceived threat, cues to action and likelihood of action predicted the behavioural outcome of anticipated time to symptomatic presentation. A baseline model was created, with the same parameters used for configural models for analysis of invariance. The increased risk and population risk data were analysed simultaneously in a configural model, and constraints were then applied to the parameters to test invariance in loadings and structure across groups (see Table 3). The invariance tests examined equivalence of model parameters (intercept, regression coefficients, means, covariance and residuals) between the two risk groups.

Fit of the SEM models was determined from five fit indices: (1) chi-square (CMIN) not significant at the .05 level of significance indicates a model with good fit [[38](#_ENREF_38)]; (2) relative chi-square (CMIN/df) with a ratio within 3:1 indicates good fit [[39](#_ENREF_39)]; (3) a comparative fit index (CFI) and (4) Tucker-Lewis Index (TLI) greater than .95 indicates a model with good fit [[40](#_ENREF_40)], (5) a standardised root-mean square error of approximation (RMSEA) close to .06 indicates good fit [[40](#_ENREF_40)].

# Results

Psychological characteristics for both risk groups are provided in Table 2. All comparisons were statistically significantly different. Women at increased risk anticipated longer presentation times (p<.001), had less confidence in symptom detection (p<.01), higher perceived susceptibility (p<.001), more personal experience with ovarian cancer (p<.001), lower symptom knowledge (p<.001) and higher ovarian cancer worry (p<.001) compared to the population risk women. The population risk women had significantly better symptom knowledge than the increased risk sample. Under half of the increased risk sample (n=115, 40.8%) anticipated presenting immediately after noticing a possible ovarian cancer symptom, with 50.8% (n=507) of the population risk women anticipating presenting immediately. The most frequently recognised symptom was persistent abdominal bloating (n =247, 88.5%) by increased risk women, and vaginal bleeding after the menopause (n= 912, 92.3%) by the population risk women (see Figure 1 for all symptoms). Passing more urine than usual was least recognised by both the increased (n=76, 27.6%) and population risk women (n= 334, 37.9%).

***Structural Equation Models***

*Structural equation model for total sample*

The full SEM for the total sample is shown in Figure 2. The goodness of fit statistic was significant (_x_^2^=115.68, df=11, p<.05), indicating a poor fit. Fit indices were CFI=.90 and RMSEA=.09, indicating marginal good fit, with TLI=.66 and relative chi-square *x*^2^/df= 10.52 indicating poor fit (see Supplement 1) for correlation matrix of model variables). The constructs of the HBM predicted 6% of variance in anticipated presentation.

Perceived susceptibility (β = 85, *p* <.001) and ovarian cancer worry (β = .45, *p* <.001) were both significant indicators of perceived threat in the measurement model (see Figure 2). The correlation between perceived threat and anticipated presentation was not significant (β = -.01, *p* >.05). The likelihood of action construct, which consists of perceived benefits and barriers, was negatively correlated with anticipated presentation, indicating that perceiving more benefits than barriers was associated with reduced presentation times (β = -.25, *p* <.001). Knowledge was not correlated with perceived threat (β = .01, *p* >.05); but was positively correlated with likelihood of action (β = .15, *p* <.05) and confidence in symptom detection (β = .24, *p* <.001). Confidence in symptom detection was negatively correlated with perceived threat (β = -.10, *p* <.01) and positively correlated with likelihood of action (β = .14, *p* <.001).

A test of invariance was carried out to identify differences in fit for the measurement model between the increased and population risk groups (see Table 3). Goodness of fit statistics for the configural model were *x*^2^(118.28), df=24, p<.001, *x*^2^/df= 4.93, CFI=.77, RMSEA=.05. The difference in chi-square indicated invariance for Model 1, indicating that when the structural weights (i.e., path coefficients) were constrained across the two groups there was no significant difference from the configural model. When other constraints were successively added (intercepts, means, covariances, residuals, see Models 2-5 in Table 3) there was a significant difference between models 2-5 and the configural model. Due to the invariance, multi-group analysis was conducted to identify model differences between the two risk groups. The results of the increased risk SEM and the population risk SEM are presented together in Figure 3.

*Determinants of anticipated presentation in increased risk sample*

The goodness of fit statistic was significant at the .05 level (_x_^2^=23.54, df=12, p<.05), indicating bad fit. The relative chi-square (_x_^2^/df=1.96) was under the recommended 3:1 range and indicated good fit. The CFI=.92 indicated marginal good fit, RMSEA=.06, good fit, and TLI=.76 a bad fit. The constructs of the HBM predicted 14% of variance in anticipated presentation for the increased risk group (see Figure 3). The observed relationships between the variables in the increased risk model are provided in the correlation matrix in Supplement 1. Figure 3 shows that perceived threat was determined by perceived susceptibility (β = .35, *p* <.001) and ovarian cancer worry (β = .98, *p* <.001), with both of these variables significant indicators of perceived threat in this measurement model. Perceived threat was negatively associated with anticipated presentation in this group (β = -.18, *p* <.01). Confidence in symptom detection was negatively associated with perceived threat (β = -.15, *p* <.05). Knowledge was not correlated with either perceived threat (β = .10, *p* >.05) or likelihood of action (β = .11, *p* >.05). However, a positive covariance of knowledge with confidence in symptom detection was observed (β = .31, *p* <.001). Age was negatively correlated with perceived threat (β = -.18, *p* <.01) and positively correlated with likelihood of action (β = .23, *p* <.001). Personal experience was not correlated with perceived threat (β = .06, *p* >.05), but those with personal experience had significantly higher confidence in symptom detection (β = .18, *p* <.01). Perceiving more benefits than barriers was associated with earlier anticipated presentation (β = -.34, *p* <.001).

*Determinants of anticipated presentation in population risk sample*

The goodness of fit statistic was significant at the .05 level (_x_^2^=26.31, df=12, p<.05), indicating a bad fit. The relative chi-square (_x_^2^/df=2.19) was under the recommended 3:1 range that indicates good fit. Other fit indices were CFI=.92 and RMSEA=.04, indicating marginally good fit, with TLI=.72 indicating a bad fit. The constructs of the HBM predicted 3% of variance in anticipated presentation for the population risk group (see Figure 3). The relationships between the variables used in the general population model are provided in the correlation matrix in Supplement 1. In the population risk group, perceived susceptibility was a determinant of perceived threat (β = .64, *p* <.05), but worry was not (β = .36, *p* >.05). The correlation between perceived threat and anticipated presentation was not significant in this group (β = -.03, *p* >.05). Confidence in symptom detection was associated with perceiving more benefits than barriers to presentation (β = .17, *p* <.001). The correlation between knowledge and likelihood of action was positive (β = .10, *p* <.01), with knowledge associated with perceiving more benefits than barriers to presenting. Perceiving more benefits than barriers to presentation was associated with earlier anticipated presentation (β = -.17, *p* <.001).

**Discussion**

In the absence of routine ovarian cancer screening, promoting help-seeking in the presence of symptoms is a potential route to early diagnosis [[28](#_ENREF_28)]. The current study explored determinants of anticipated symptomatic presentation in a sample of women comprising two risk populations. Women at increased risk had higher levels of worry, perceived susceptibility, and a greater degree of personal experience of ovarian cancer, and lower knowledge, and had longer anticipated time to symptom presentation than the general population sample. This is the first study to compare data on levels of worry and perceived susceptibility in a general population and increased risk sample, and to examine how worry and perceived susceptibility interact with help-seeking intentions. Findings suggest that health beliefs relating to ovarian cancer are related to risk status. Determinants of earlier symptomatic presentation that were common to both groups included high perceived susceptibility, high confidence in symptom detection, high symptom knowledge and perceiving more benefits than barriers to presentation. However, the cancer worry component of perceived threat was a unique predictor in the increased risk group. The current findings support the need for an ovarian cancer awareness intervention that emphasises the perceived benefits of symptom presentation and minimises perceived barriers, and that increases confidence in symptom detection whilst managing worry.

Evidence suggests that higher perceived threat increases the likelihood of engaging in behaviour that is likely to manage or reduce this threat [[17](#_ENREF_17)]. The present findings add to this by suggesting that, in certain patient groups, that worry may be a greater motivator of earlier presentation than the susceptibility component of perceived threat. Perceived threat was observed to comprise both worry and susceptibility components in women at increased risk, but only susceptibility aspects in the population risk sample. The appraisal process through which people generate a sense of personal susceptibility could therefore be an important target for future research on cancer awareness, helping researchers to better conceptualise “delay” from the patient’s perspective [[41](#_ENREF_41)]. It may be that emotional processes are more influential in certain patient groups (such as those at increased risk) and therefore may need consideration when conceptualising the symptom appraisal interval. The current findings could suggest that women at increased risk have perceived susceptibility and ovarian cancer worry determinants in this appraisal process. It should be noted that perceived susceptibility partly reflected a true element (as the increased risk women genuinely were at risk), highlighting that perceived susceptibility encompasses many elements (i.e knowledge, construal of risk and emotions) that need to be better understood.

The association between earlier anticipated symptom presentation and worry in increased risk women in the current study complements research on familial ovarian cancer screening uptake, where worry has been shown to be a key determinant of screening uptake [[18](#_ENREF_18), [23](#_ENREF_23)]. The findings relating to worry could also have practical implications for healthcare professionals who should be aware of the potential for heightened cancer worry when consulting with people at increased risk. Results in the current study indicated that women from the general population had higher symptom knowledge and anticipated presenting in shorter time frames than the increased risk sample. The observed difference could reflect that women at increased risk in the UK have previously relied on screening as their main detection strategy and therefore place less emphasis on symptom knowledge and symptomatic presentation. The type of knowledge women have about ovarian cancer will also vary across the lifespan according to maturational and experiential factors (e.g., reproductive change).

Factor analysis of the HBM scales showed the same pattern of underlying constructs for the two risk groups, with the exception of barriers. For the increased risk sample, barriers were differentiated by fear of the discovery of ovarian cancer, whereas for the population risk group the practical barriers reflecting time constraints were more salient. This differentiation is an important finding and could have implications for education and awareness about ovarian cancer. The results could suggest that regardless of risk status, all women could benefit from ovarian cancer symptom information and education about presentation times. Therefore an intervention with tailored content that addresses the specific needs of women at increased risk could be embedded within an inclusive tool containing core symptom information that addresses generic educational needs.

A greater proportion of variance in anticipated presentation was predicted for the increased risk group (14%) than for the population risk group (3%). Tests of invariance indicated that the difference between the two groups was due to differences in the magnitude of path coefficients in the model, rather than differences in levels of predictors (e.g. mean susceptibility). The path differences suggest that health beliefs in women at increased risk are determined by perceived threat, with emotional representations of this latent variable important in this population. The varying model fit could be explained in terms of the study populations. The model may not fit the general population so well because it does not represent the health beliefs of this group, or their notion of threat, as well as it does for the increased risk group. The HBM proposes that when faced with a potential health threat, people consider their susceptibility to and the severity of the health threat when deciding whether to act, [[17](#_ENREF_17)], with such considerations more salient in those at increased risk. This could also explain the greater proportion of variance in anticipated presentation that was accounted for by the model in women at increased risk.

The cross-sectional study design and use of intent-to-present have implications regarding the temporal stability and interpretation of the current findings. Although causality cannot be inferred, the current research provides an important contribution as it has identified health beliefs in different risk populations in relation to anticipated symptomatic presentation. It should also be noted that the current findings may reflect more about cognitive appraisal of what to do in the presence of symptoms (intentions) as opposed to actual behaviour [[42](#_ENREF_42)], with actual behaviour possibly less prompt than intentions [[12](#_ENREF_12)]. In addition to the use of intentions, the use of a dichotomous variable for anticipated presentation could obscure nuances in this variable. However, the cut-off of ‘immediate presentation’ versus ‘any delay’ was chosen in the absence of clinical consensus regarding the optimal time to present with ovarian cancer symptoms, and recognising that the presence of delay may be more important than the degree of delay [[43](#_ENREF_43), [44](#_ENREF_44)].

Symptom knowledge scores were aggregated in this study, whereas a deeper understanding may be gained if knowledge of individual symptoms, such as specific versus non-specific symptoms, was examined. However, the current sample size was insufficient to permit such fine-grained analysis. In addition, the symptom question does not inform about the processes women may go through when appraising and interpreting a symptom, or if indeed the participants are simply guessing whether symptoms were indicative of ovarian cancer.

Model fit has previously been discussed, but group differences should also be noted as a possible explanation of differences observed in the SEM. The demographic profiles of the two samples, for example variables including age and education level rather than cancer awareness could explain the observed effects. The potential lack of sample representativeness is also acknowledged, as the increased risk women were recruited from those who had participated in a screening evaluation study. These women may therefore have different levels of ovarian cancer worry and symptom knowledge than women who did not take part. The limitations of sampling methods are also acknowledged, since the cases and controls were not drawn from the same population [[45](#_ENREF_45)]. A further concern is the different sampling methods that were used. The increased genetic risk sample was an opportunity sample whilst the general population sample was a population representative sample. The demographic profiles of the two samples could explain differences observed, therefore it could be variables including age and education level, rather than cancer awareness that caused the observed effects.

***Conclusions***

The current research has developed an understanding of anticipated presentation with ovarian cancer symptoms. In both risk populations, raising awareness of the benefits of presenting with symptoms and dispelling the barriers is important. Prospective research that examines actual behaviour and that disentangles causal direction is an important next step in this research field. This study highlights the need to develop an ovarian symptom information tool in which content is tailored according to ovarian cancer risk.

**List of abbreviations:**

HBM – Health Belief Model; SEM – Structural equation modelling

**Ethics approval and consent to participate:** The study received ethical approval from Cardiff University. Written informed consent was obtained from all individual participants included in the study.

**Consent for publication:** Not applicable.

**Availability of Data and Materials:** The dataset supporting conclusion of this article are available upon request to the lead author. Data requests for anonymised study data will be reviewed by the study team. Requests should be made to the lead author.

**Competing interests:** Authors Stephanie Smits, Jacky Boivin, Usha Menon and Kate Brain declare that they have no conflict of interest.

**Funding**: SS was funded through a PhD studentship which received 50% funding support from the Medical Research Council and 50% from Cardiff University. The funders had no role in the design of the study, data collection, analysis, data interpretation or writing the manuscript.

**Authors’ contributions:** SS, KB, JB and UM conceived the study design. SS analysed data with the supervision of KB and JB. SS drafted the manuscript. KB, JB and UM participated in drafting the manuscript. All authors read and approved the final manuscript.

**Acknowledgments:** We would like to thank the women who took part in the study.

# References

1. Lataifeh I, Marsden DE, Robertson G, Gebski V, Hacker NF: **Presenting symptoms of epithelial ovarian cancer**. *Aust N Z J Obstet Gynaecol* 2005, **45**(3):211-214.

2. Goff BA, Mandel LS, Drescher CW, Urban N, Gough S, Schurman KM, Patras J, Mahony BS, Andersen MR: **Development of an ovarian cancer symptom index - Possibilities for earlier detection**. *Cancer* 2007, **109**(2):221-227.

3. Smith RA, Cokkinides V, Eyre HJ: **American Cancer Society Guidelines for the Early Detection of Cancer, 2005**. *CA Cancer J Clin* 2005, **55**(1):31-44; quiz 55-36.

4. Richards MA: **The size of the prize for earlier diagnosis of cancer in England**. *Br J Cancer* 2009, **101**:S125-S129.

5. Evans J, Ziebland S, McPherson A: **Minimizing delays in ovarian cancer diagnosis: an expansion of Andersen's model of ‘total patient delay’**. *Fam Pract* 2007, **24**(1):48-55.

6. Kirwan JM, Tincello DG, Herod JJ, Frost O, Kingston RE: **Effect of delays in primary care referral on survival of women with epithelial ovarian cancer: retrospective audit**. *Bmj* 2002, **324**(7330):148-151.

7. Hamilton W, Peters TJ, Bankhead C, Sharp D: **Risk of ovarian cancer in women with symptoms in primary care: population based case-control study**. *Bmj* 2009, **339**:b2998-b2998.

8. Austoker J: **Diagnosis of ovarian cancer in primary care**. *Bmj* 2009, **339**:583-589.

9. Menon U, Gentry-Maharaj A, Hallett R, Ryan A, Burnell M, Sharma A, Lewis S, Davies S, Philpott S, Lopes A: **Sensitivity and specificity of multimodal and ultrasound screening for ovarian cancer, and stage distribution of detected cancers: results of the prevalence screen of the UK Collaborative Trial of Ovarian Cancer Screening (UKCTOCS)**. *The Lancet Oncology* 2009, **10**(4):327-340.

10. Jacobs IJ, Menon U, Ryan A, Gentry-Maharaj A, Burnell M, Kalsi JK, Amso NN, Apostolidou S, Benjamin E, Cruickshank D: **Ovarian cancer screening and mortality in the UK Collaborative Trial of Ovarian Cancer Screening (UKCTOCS): a randomised controlled trial**. *The Lancet* 2015, **387**:945-956.

11. Prat J: **Staging classification for cancer of the ovary, fallopian tube, and peritoneum**. *International Journal of Gynecology & Obstetrics* 2014, **124**(1):1-5.

12. Robb K, Stubbings S, Ramirez A, Macleod U, Austoker J, Waller J, Hiom S, Wardle J: **Public awareness of cancer in Britain: a population-based survey of adults**. *Br J Cancer* 2009, **101**:S18-S23.

13. Forbes LJL, Simon AE, Warburton F, Boniface D, Brain KE, Dessaix A, Donnelly C, Haynes K, Hvidberg L, Lagerlund M: **Differences in cancer awareness and beliefs between Australia, Canada, Denmark, Norway, Sweden and the UK (the International Cancer Benchmarking Partnership): do they contribute to differences in cancer survival&quest**. *Br J Cancer* 2013, **108**(2):292-300.

14. Simon AE, Waller J, Robb K, Wardle J: **Patient Delay in Presentation of Possible Cancer Symptoms: The Contribution of Knowledge and Attitudes in a Population Sample from the United Kingdom**. *Cancer Epidem Biomar* 2010, **19**(9):2272-2277.

15. Lockwood-Rayermann S, Donovan HS, Rambo D, Kuo CWJ: **Original Research: Women's Awareness of Ovarian Cancer Risks and Symptoms**. *AJN The American Journal of Nursing* 2009, **109**(9):36-45.

16. Low EL, Waller J, Menon U, Jones A, Reid F, Simon AE: **Ovarian cancer symptom awareness and anticipated time to help-seeking for symptoms among UK women**. *Journal of Family Planning and Reproductive Health Care* 2013, **39**(3):163-171.

17. Rosenstock I, Strecher V, Becker M: **Social learning theory and the health belief model**. *Health Education & Behavior* 1988, **15**(2):175-183.

18. Schwartz MD, Lerman C, Miller SM, Daly M, Masny A: **Coping disposition, perceived risk, and psychological distress among women at increased risk for ovarian cancer**. *Health Psychology* 1995, **14**(3):232-235.

19. Brain K, Norman P, Gray J, Mansel R: **Anxiety and adherence to breast self-examination in women with a family history of breast cancer**. *Psychosomatic medicine* 1999, **61**(2):181-187.

20. Norman P, Brain K: **An application of an extended health belief model to the prediction of breast self-examination among women with a family history of breast cancer**. *Br J Health Psychol* 2005, **10**(Pt 1):1-16.

21. Glanz K, Rimer BK, Viswanath K: **Health behavior and health education: theory, research, and practice**: John Wiley & Sons; 2008.

22. Hay JL, Buckley TR, Ostroff JS: **The role of cancer worry in cancer screening: a theoretical and empirical review of the literature**. *Psycho‐Oncology* 2005, **14**(7):517-534.

23. Andersen MR, Peacock S, Nelson J, Wilson S, McIntosh M, Drescher C, Urban N: **Worry about ovarian cancer risk and use of ovarian cancer screening by women at risk for ovarian cancer**. *Gynecol Oncol* 2002, **85**(1):3-8.

24. Kash KM, Holland JC, Halper MS, Miller DG: **Psychological distress and surveillance behaviors of women with a family history of breast cancer**. *J Natl Cancer Inst* 1992, **84**(1):24-30.

25. Lerman C, Daly M, Masny A, Balshem A: **Attitudes about genetic testing for breast-ovarian cancer susceptibility**: Journal of Clinical Oncology. 12 (4) 843-850; 1994.

26. Brain K, Parsons E, Bennett P, Cannings‐John R, Hood K: **The evolution of worry after breast cancer risk assessment: 6‐year follow‐up of the TRACE study cohort**. *Psycho‐Oncology* 2011, **20**(9):984-991.

27. Evans RE, Morris M, Sekhon M, Buszewicz M, Walter FM, Waller J, Simon AE: **Increasing awareness of gynaecological cancer symptoms: a GP perspective**. *Brit J Gen Pract* 2014, **64**(623):e372-e380.

28. Brain KE, Smits S, Simon AE, Forbes LJ, Roberts C, Robbé IJ, Steward J, White C, Neal RD, Hanson J: **Ovarian cancer symptom awareness and anticipated delayed presentation in a population sample**. *BMC Cancer* 2014, **14**(1):171-181.

29. CRUK: **Cancer research UK ovarian cancer UK statistics.** 2011, **accessed January 2013**(available at: <http://publications.cancerresearchuk.org/downloads/Product/cs_pdf_ovarian_march_2011.pdf)>.

30. Brain KE, Lifford KJ, Fraser L, Rosenthal AN, Rogers MT, Lancastle D, Phelps C, Watson EK, Clements A, Menon U: **Psychological Outcomes of Familial Ovarian Cancer Screening: No Evidence of Long-Term Harm**. *Gynecol Oncol* 2012, **127**(3):556-563.

31. Rosenthal AN, Fraser L, Manchanda R, Badman P, Philpott S, Mozersky J, Hadwin R, Cafferty FH, Benjamin E, Singh N: **Results of annual screening in phase I of the United Kingdom familial ovarian cancer screening study highlight the need for strict adherence to screening schedule**. *Journal of Clinical Oncology* 2012:JCO. 2011.2039. 7638.

32. Rizzo L, Brick JM, Park I: **A minimally intrusive method for sampling persons in random digit dial surveys**. *Public Opinion Quarterly* 2004, **68**(2):267-274.

33. Tyndel S, Austoker J, Henderson BJ, Brain K, Bankhead C, Clements A, Watson EK: **What is the psychological impact of mammographic screening on younger women with a family history of breast cancer? Findings from a prospective cohort study by the PIMMS Management Group**. *Journal of Clinical Oncology* 2007, **25**(25):3823-3830.

34. Andersen MR, Drescher CW, Zheng Y, Bowen DJ, Wilson S, Young A, McIntosh M, Mahony BS, Lowe KA, Urban N: **Changes in cancer worry associated with participation in ovarian cancer screening**. *Psycho‐Oncology* 2007, **16**(9):814-820.

35. Lerman C, Trock B, Rimer BK, Jepson C, Brody D, Boyce A: **Psychological side effects of breast cancer screening**. *Health Psychology* 1991, **10**(4):259-267.

36. Simon AE, Wardle J, Grimmett C, Power E, Corker E, Menon U, Matheson L, Waller J: **Ovarian and cervical cancer awareness: development of two validated measurement tools**. *Journal of Family Planning and Reproductive Health Care* 2012, **38**(3):167-174.

37. Department of Health: **Department of Health key messages for ovarian cancer for health professionals.** . 2009, **[accessed January 2013]**(available at: <http://www.ovarian.org.uk/media/8815/ovarian_cancer_kms_final.pdf)>.

38. Byrne BM: **Structural equation modeling with AMOS: Basic concepts, applications, and programming**: CRC Press; 2009.

39. Kline RB: **Principles and practice of structural equation modeling**: Guilford press; 2011.

40. Hu Lt, Bentler PM: **Cutoff criteria for fit indexes in covariance structure analysis: Conventional criteria versus new alternatives**. *Structural Equation Modeling: A Multidisciplinary Journal* 1999, **6**(1):1-55.

41. Andersen R, Vedsted P, Olesen F, Bro F, Søndergaard J: **Patient delay in cancer studies: a discussion of methods and measures**. *BMC Health Serv Res* 2009, **9**(1):189-196.

42. Sheeran P: **Intention—behavior relations: A conceptual and empirical review**. *European review of social psychology* 2002, **12**(1):1-36.

43. Macleod U, Mitchell ED, Burgess C, Macdonald S, Ramirez AJ: **Risk factors for delayed presentation and referral of symptomatic cancer: evidence for common cancers**. *Br J Cancer* 2009, **101**:S92-S101.

44. Ramirez AJ, Westcombe AM, Burgess CC, Sutton S, Littlejohns P, Richards MA: **Factors predicting delayed presentation of symptomatic breast cancer: a systematic review**. *Lancet* 1999, **353**(9159):1127-1131.

45. Mann C: **Observational research methods. Research design II: cohort, cross sectional, and case-control studies**. *Emergency Medicine Journal* 2003, **20**(1):54-60.

**Figure legends**

Figure 1. Recognition of individual ovarian cancer symptoms for both risk groups

Legend: valid % presented in cases where data were missing

Figure 2. SEM for HBM applied to anticipated presentation with potential ovarian cancer symptoms for all participants.

Legend: The SEM investigates whether the HBM variables of individual perceptions (perceived susceptibility and worry), modifying factors (age, knowledge, confidence), perceived threat, cues to action (personal experience) and likelihood of action (perceived benefits minus barriers) predicted the behavioural outcome of anticipated presentation for all participants.

Values displayed are standardised regression weights ( ), covariances ( ) and percentage of variance accounted for. Squares represent observed variables, and circles represent unobserved variables. ns= not statistically different.*p<.05. **p<.01, ***p<.001. *(SEM = structural equation model, HBM = health belief model)*

Figure 3. SEM for HBM applied to anticipated presentation with potential ovarian cancer symptoms for both groups.

Legend: The SEM investigates whether the HBM variables of individual perceptions (perceived susceptibility and worry), modifying factors (age, knowledge, confidence), perceived threat, cues to action (personal experience) and likelihood of action (perceived benefits minus barriers) predicted the behavioural outcome of anticipated presentation for both groups.

Increased risk group is the top coefficient (bold and italics), and the general population group is the bottom coefficient (not bold/not italics). Values displayed are standardised regression weights ( ), covariances ( ) and percentage of variance accounted for. Squares represent observed variables, and circles represent unobserved variables. ns= not statistically significant.*p<.05. **p<.01, ***p<.001. *(SEM = structural equation model, HBM = health belief model)*

**Additional files**

File name: Supplement 1

Title of data: Correlation matrix for variables in the structural equation models

Description of data: correlation matirx, means and standard deviations for variables in the three structural equation models
